# LARP1 predict the prognosis for early-stage and AFP-normal hepatocellular carcinoma

**DOI:** 10.1186/1479-5876-11-272

**Published:** 2013-10-26

**Authors:** Chan Xie, Li Huang, Shibin Xie, Dongying Xie, Genlin Zhang, Peipei Wang, Liang Peng, Zhiliang Gao

**Affiliations:** 1 Department of Infectious Diseases, The Third Affiliated Hospital of Sun Yat-Sen University, 600# TianHe Road, Guangzhou 510630, Guangdong Province, China; 2 Key Laboratory of Tropical Disease Control, Ministry of Education, Sun Yat-Sen University, Guangzhou, Guangdong Province, China; 3 Department of nephrology, The Third affiliated hospital of Guangzhou medical university, Guangzhou, China

**Keywords:** LARP1, Hepatocellular carcinoma, AFP, Prognosis

## Abstract

**Background:**

The La-related protein 1 (LARP1) has been found to be a RNA binding protein and was related to spermatogenesis, embryogenesis and cell-cycle progression. The aim of this study was to investigate the prognostic value of LARP1 in hepatocellular carcinoma (HCC).

**Methods:**

LARP1 expression was examined in 15 HCC cell lines and 272 clinical specimens using real-time PCR, immunohistochemistry (IHC) and western blot analysis (WB). LARP1 expression was also studied in 6 paired HCC lesions and the adjacent non-cancerous tissue samples. Statistical analyses were applied to derive association between LARP1 expression scores and clinical characters as well as patient survival.

**Results:**

mRNA and protein levels of LARP1 were higher in HCC cell lines and HCC lesions than in normal liver epithelial cells and the paired adjacent noncancerous tissues. LARP1 expression was correlated to survival time, vital status, tumor size and Child-Pugh score. Overall survival analysis showed HCC patients with high LARP1 expression level had lower survival rate (*P* < 0.01). Importantly, this correlation remained significant in patients with early-stage HCC or with normal serum AFP level.

**Conclusions:**

LARP1 protein may represent a promising biomarker for predicting the prognosis of HCC, including in early-stage and AFP-normal patients.

## Introduction

Hepatocellular carcinoma (HCC) is one of the most commonly diagnosed cancers and the third most common cause of cancer mortality worldwide [1]. Despite great advances in surgical and medical management of the disease, relapse and metastasis are frequently observed in the clinic, and the long-term prognosis of patients with HCC is unsatisfactory. The development and progression of HCC is a multistage process involves molecular pathways and genetic abnormal changes [2]. No specific signature of liver cancer gene expression has been reported to allow for patient-tailored therapy strategies. Hence, it is of great clinical value to further identify effective early markers for the diagnosis and prognosis of the disease as well as novel therapeutic targets.

The progression of HCC is thought to involve the deregulation of genes that are critical to cellular processes such as cell cycle control, cell growth, apoptosis, and cell migration and spreading. Clinically, prediction of prognosis plays an essential role in the assessment of HCC patients and optimal therapeutic options. As an increasing number of early-stage, small HCC nodules (< 3 cm) in patients with normal serum level of alpha fetal protein (AFP), a most commonly used diagnostic biomarker for HCC, prediction of prognosis of these patients represents a major challenge in the clinic [2]. While tremendous effort has been made in identifying effective indicators for the prognostic prediction of these HCC, the availability of clinically applicable biomarkers remains limited. La-related protein 1 (Larp1) was first described in Drosophila and shown to be required for spermatogenesis, embryogenesis and cell cycle progression [3-5]. Phenotypically, loss of Larp1 causes an apoptosis, mitotic arrest and reduction in formation of migratory lamellipodia in cell [6]. These data raise exciting questions about the role of Larp1 in mRNA translation and cell migration, and suggest Larp1 may have relevance in a number of conditions in which these processes are disrupted, such as in protein production and cancer.

In our current report, immunohistochemical analysis was performed to investigate the potential prognostic utility of LARP1 in a validation cohort of HCC in comparison with non-neoplastic liver tissues, including HCC patients with normal serum AFP level of early stage. Our data suggest that LARP1 might represent a valuable prognostic biomarker for HCC.

## Materials and methods

### Cell lines

HCC cell lines, including QGY-7703, HCCC-9810, PLC/PRF5, SMMC-7721, Hep3B, HepG2, HuH7,HepG-2215, Bel-7402, QGY-7701, Bel-7404, HCCLM3, HCCLM6, MHCC97H and MHCC97L, were grown in Dulbecco’s modified Eagle’s medium (DMEM) (Invitrogen, Carlsbad, CA) supplemented with 10% fetal bovine serum (FBS) (Invitrogen). Immortalized normal liver epithelial cells, THLE3, were maintained in bronchial epithelial growth medium (Lonza Cologne GmbH, Walkersville, MD), with 5 ng/ml epidermal growth factor, 70 ng/ml phosphoethanolamine and 10% FBS, at 37°C in a humidified atmosphere containing 5% CO_2_.

### Patient cohorts

A total of 272 cases of paraffin-embedded HCC samples which had been clinically and histologically diagnosed at the Sun Yat-Sen University Cancer Center (Guangzhou, China) from 1996 to 2004. Six fresh HCC tissue samples, together with their paired adjacent non-cancerous tissues from each patient in the third affiliated hospital of Guangzhou medical university, were collected from HCC curative resection surgery from 2011 to 2012, snap frozen and stored at -80°C until use for experimental purposes. For the use of these clinical materials for research purposes, prior patients’ consents and approval from the Institutional Research Ethics Committee were obtained. This cohort of patients with HCC included 244 (89.7%) men and 28 (10.3%) women, with a median age of 49 years, and their clinico-pathological characteristics are summarized in Table 1. Tumor stages were defined according to the 2002 American Joint Committee on Cancer/International Union against Cancer tumor/lymph node metastasis/distal metastasis (TNM) classification system. Hepatitis B virus (HBV) infection was diagnosed when HBV surface antigen (HBsAg) was detected by ELISA in the serum. In addition, for Western blot analysis, three normal liver tissues were obtained from the patients undergoing resection of hepatic hemangiomas at the Department of Hepatobiliary Surgery in 2011, the First Affiliated Hospital of Sun Yat-sen University, in accordance with rules and regulations concerning ethical issues on research use of human subjects in China. Follow-up visits were scheduled postoperatively at intervals of one month for six months, bimonthly for six months, quarterly for six months, and semiannually for life.

**Table 1:** Clinicopathological characteristics of clinical samples and expression of LARP1 in liver cancer

**Characteristics**		**No. Patients**	**%**
Age (years)	≤50	159	41.5
	>50	113	58.5
Gender	Male	244	89.7
	Female	28	10.3
TNM classification	I	16	5.9
	II	195	71.7
	III	61	22.4
HBsAg	Positive	225	87.5
	Negative	32	12.5
AFP	≥400 ng/ml	100	38.0
	<400 ng/ml	163	62.0
Tumor size	>3 cm	228	84.4
	≤3 cm	42	15.6
Tumor number	>1	93	34.4
	= 1	177	65.6
Child-pugh class	A	264	97.1
	B	8	2.9
Vital status (at follow-up)	Alive	152	55.9
	Death due to liver cancer	120	44.1
Expression of LARP1	High expression	101	37.1
	Low expression	171	62.9

### RNA extraction, reverse transcription (RT) and real-time PCR

Total RNA from cultured cells was extracted using the Trizol reagent (Invitrogen, Carlsbad, CA) as the manufacturer instructed. cDNAs were amplified and quantified in ABI Prism 7500 Sequence Detection System (Applied Biosystems, Foster City, CA) using dye SYBR Green I (Invitrogen, Carlsbad, CA). The LARP1 primers designed using the Primer Express v 2.0 software (Applied Biosystems) are provided as following: forward: 5′- GCTGTTTAGGAACAGCTGCC -3′ and reverse: 5′- CCACAGGTGACAGGGAGAAG-3′. Expression data were normalized to the geometric mean of housekeeping gene GAPDH (forward: 5′-ACCACAGTCCATGCCATCAC-3′ and reverse: 5′-TCCACCACCCTG TTGCTGTA -3′) to control the variability in expression levels and calculated as 2^-[(*C*^*t*^of LARP1) – (*C*^*t*^of *GAPDH*)]^, where C_t_ represents the threshold cycle for each transcript.

### Western blot

Total protein was prepared using the cell total protein extraction kits according to the manufacturer’s instruction (Millipore, Billerica, MA). Western blot was performed according to standard methods, using an anti-LARP1 antibody (Abcam, UK). Blotted membranes were stripped and re-probed with an anti-GAPDH antibody (Sigma, Saint Louis, MI) as a loading control.

### Immunohistochemistry (IHC)

The IHC procedure for LARP1 and scoring of LARP1 expression were performed as previously reported [7]. IHC staining was quantitatively analyzed with the AxioVision Rel.4.6 computerized image analysis system assisted with the automatic measurement program (Carl Zeiss, Oberkochen, Germany). Briefly, the stained sections were evaluated at 200x magnification, and ten representative staining fields of each section were analyzed to produce Mean Optical Density value (MOD), which represented the strength of staining signals as measured per positive pixels. The MOD data were statistically analyzed using *t*-test to compare the average MOD difference between different groups of tissues. An optimal cutoff value was identified: the staining index score of > 6 was used to define tumors as high LARP1 expression and ≤ 6 as low expression of LARP1.

### Gene Expression Omnibus database analysis

Gene Expression Omnibus database (GEO GSE25097) was used in the analysis of relationship of LARP1 expression and HCC.

### Statistical analysis

All statistical analyses were carried out using the SPSS v. 13.0 statistical software packages (SPSS, Chicago, IL). Spearman’s correlation test was applied to analyze the correlation between LARP1 expression and clinic pathological characteristics. Survival curves were plotted by the Kaplan-Meier method and compared using the log-rank test. Independent prognostic factors were estimated by the Cox proportional hazards stepwise regression model. We first used SPSS statistic software to perform the Univariate analysis of each variance and found the factors which is significant different in the two groups and then perform the multivariate analysis. All *P* values were two-sided. A *P* value of less than 0.05 was considered statistically significant in all cases.

## Results

### Upregulation of LARP1 in HCC cell lines and liver cancer lesions

**Figure 1:**
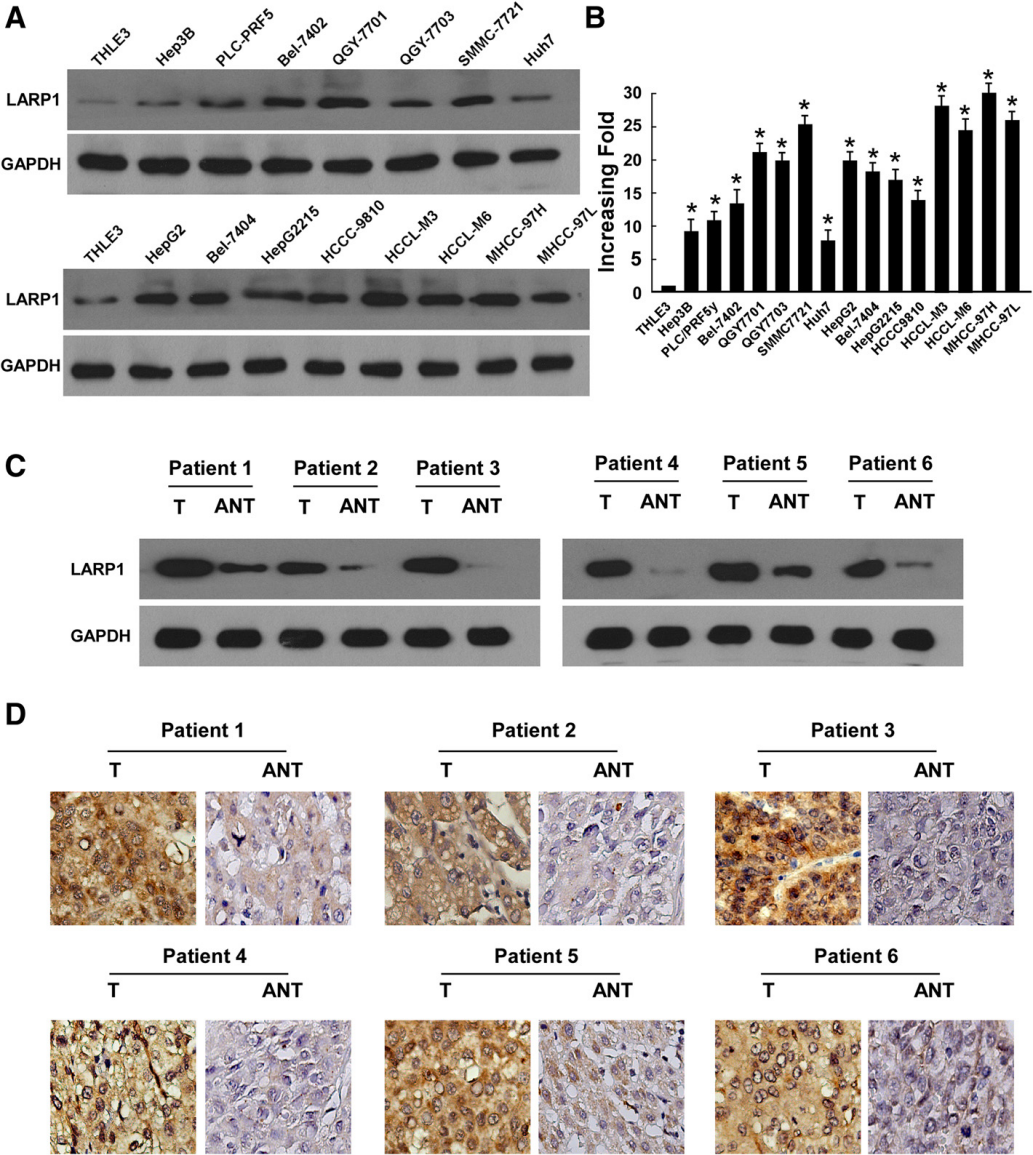
**Expression of LARP1 is elevated in HCC. (A-B)** Expression of LARP1 protein **(A)** and mRNA **(B)** in normal human liver epithelial cells (THLE3) and cultured liver cancer cell lines. GAPDH was used as a loading control. **(C-D)** Western blot **(C)** and IHC **(D)** analysis of LARP1 protein in each of the primary liver cancer tissue (T) and adjacent non-cancerous tissues (ANT) samples taken from the same patient. Error bars represent SD from three independent experiments. * *P* < 0.05.

Western blot and real-time PCR analyses revealed LARP1 was highly expressed in all fifteen HCC cell lines tested than that in immortalized normal liver epithelial cells THLE3 (Figure 1A and B). To determine whether LARP1 upregulation found in liver cancer cell lines was related to clinical biochemical indicators, Western blot analysis was performed with 6 paired HCC tissues and non-cancerous tissues adjacent to HCC tumors. As shown in Figure 1C, LARP1 protein expressed was higher in all six HCC samples, displaying more than 3-fold increase of LARP1 expression as compared that in the adjacent non-cancer tissue samples. In agreement with the result of Western blot assay, immunohistochemical analysis also showed LARP1 upregulation in HCC lesions (Figure 1D).

### Association between LARP1 expression and clinical features of liver cancer

**Figure 2:**
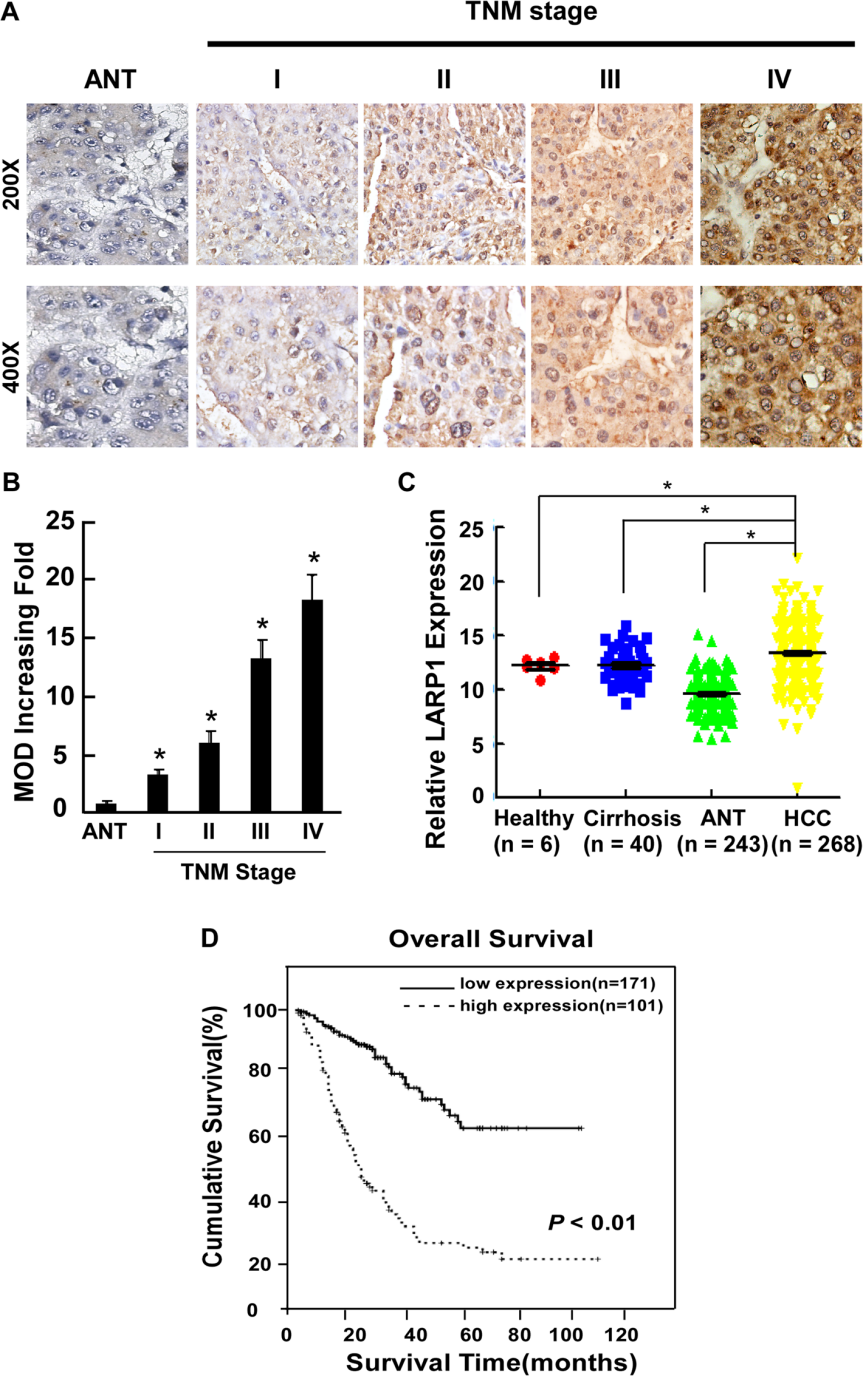
**Overexpression of LARP1 in archived HCC. (A)** Representative IHC analyses of LARP1 expression in normal liver tissue (NL) and HCC specimens of different TNM stages. Statistical quantification of the average of the mean absorbance ( MOD) values of LARP1 staining between normal liver tissue (NL) and HCC specimens of different TNM stages. **(B)** MOD of LARP1 staining increases as HCC progresses to a higher clinical stage. **(C)** Gene Expression Omnibus database (GEO GSE25097) analysis showed different mRNA expression in the indicated tissues. **(D)** Kaplan–Meier curves with univariate analyses (log-rank) for patients with low- versus high-LARP1 expression.

To determine the clinical significance of LARP1, the correlation between the clinicopathological features of HCC and LARP1 expression was investigated in a retrospective cohort of 272 HCC cases by IHC, including 16 cases of stage I (5.9%), 195 cases of stage II (71.7%) and 61 cases of stage III (22.4%) , based on the TNM staging. In the cohort, 87.5% patients had HBV infection. LARP1 expression in 272 enrolled patient samples was determined as strong in 101 cases (37.1%) and weakly positive or negative in 171 cases (62.9%) (Table 1). As shown in Figure 2A, the immunoreactivity of LARP1 was detected at variable levels and localized in the cellular nucleus and plasma. The LARP1 protein expression was generally weak in adjacent non-cancer tissue samples and early stage HCC (TNM stages I and II), but strong in later stage HCC (TNM stages III) tissues. Quantitative analysis of the IHC staining indicated that LARP1 expression in clinical stage I–III primary tumors was statistically higher than that in adjacent non-cancer tissue samples (*P* < 0.05, Figure 2B). Spearman analysis revealed correlations between LARP1 and Tumor size (*P P* < 0.05), survival time (*P* < 0.01), Child-Pugh (*P* < 0.01) and vital status (*P* < 0.01) (Table 2). No significant associations between expression of LARP1 and other clinicopathological parameters such as age, HBsAg status, tumor size and number of tumor nodules (Table 2), further suggesting a correlation of LARP1 expression with HCC clinical staging and patient survival. To confirm the result of the relationship of LARP1 with HCC, we analysis the different expression of LARP1 in a Microarray data which have been deposited in the Gene Expression Omnibus database (GEO GSE25097). This database included mRNA information of tissues of 6 healthy peoples, 40 liver cirrhosis patients, 243 non-tumor tissues from HCC patients and 268 HCC tumor tissues. The result showed that the expression of LARP1in HCC patients is higher than that in healthy person. LARP1 is highly expressed in HCC than in adjacent non-tumor tissues (Figure 2C). But the LARP1 expression of liver cirrhosis patients did not change. And the LARP1 expression of adjacent non-tumor tissues was lower in the healthy persons. The meaning of the decrees of LARP1 in adjacent non-tumor tissues is wildly unknown and more deeper research needed to clarify the mechanism of LARP1 in HCC develop.

**Table 2:** Spearman analysis of correlation between LARP1 and clinicopathological factors

**Variables**	**LARP1 expression level**	
	**Spearman Correlation**	***P *** **Value**
Survival time	- 0.317	***0.000***
Vital status	0.512	***0.000***
HBsAg	0.074	*0.226*
Age	0.001	*0.986*
TNM	0.118	*0.052*
AFP	-0.010	*0.867*
gender	-0.110	*0.070*
Tumor number	0.045	*0.458*
Tumor size	0.137	***0.023***
Child-Pugh	0.163	***0.007***

### Univariate and multivariate analyses of the prognostic power of LARP1

To identify variables with potential prognostic significance in HCC patients, univariate analysis for each variable was performed in relation to the survival time. In our univariate analysis, stepwise inclusion of variables in the model showed that significant prognostic factors included LARP1 level, tumor size, tumor number, Child-Pugh class and TNM classification. Moreover, multivariate analysis demonstrate that tumor size, tumor number, Child-Pugh class and LARP1 expression level are indeed predictive of the overall survival (OS) of HCC patients (Table 3).

**Table 3:** Univariate and multivariate analyses of various prognostic parameters in patients with liver cancer by Cox-regression analysis

	**Univariate analysis**			**Multivariate analysis**		
	**Relative risk**	**95% confidence interval**	***P***	**Relative risk**	**95% confidence interval**	***P***
Tumor size	1.061	1.024-1.101	*0.001*	1.056	1.019-1.094	*0.002*
Tumor number	1.116	1.051-1.184	*0.000*	1.151	1.075-1.232	*0.000*
LARP1	1.280	1.205-1.395	*0.000*	1.252	1.170-1.339	*0.000*
Child-Puge	1.562	1.322-1.847	*0.000*	1.219	1.013-1.466	*0.036*

### Prognostic values of LARP1 in different HCC subgroups

To further demonstrate the value of LARP1 expression in predicting survival of HCC patients, multiple analysis methods were performed in this study. Firstly, as shown in Figure 2D, Kaplan-Meier and log-rank survival tests suggested that low- and high-LARP1 expression in HCC patients were associated with different survival time, with the OS of patients expressing low LARP1 in their HCC lesions surviving much longer that those with high LARP1 expression (*P* < 0.01). Interestingly, these patients with different OS could not be distinguished by conventional AFP test. While for the whole study cohort, the OS rates at 3, 5 and 8 years, respectively, were 46%, 37% and 33%, patients with high LARP1 protein levels had a significantly lowered 3, 5, 8-year survival rate than those with low LARP1 protein levels (14%, 10%, 7% vs 68%, 57%, 57%, respectively, *P* < 0.05).

**Figure 3:**
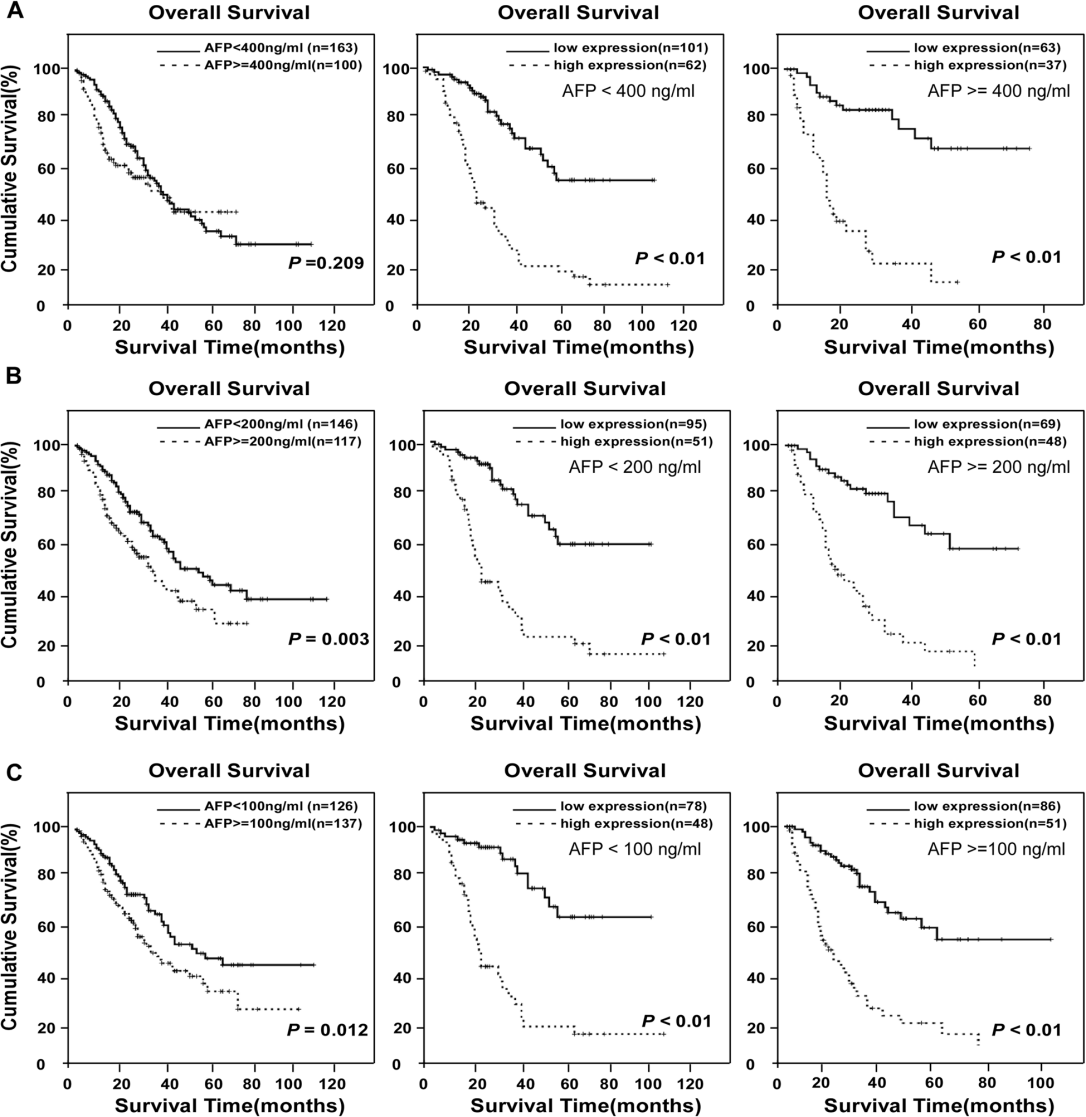
**Kaplan-Meier analysis of OS in 272 patients based on LARP1 expressions in HCC subgroups of different AFP levels. (A)** Using AFP level (400 ng/ml) as the cut-off could not separate patients with different OS rates in the study cohort (left). By contrast, LARP1 expression level predicted different OS rate in the subgroup of AFP < 400 ng/ml and AFP ≥ 400 ng/ml. **(B)** OS rate of patients with AFP <200 ng/ml and AFP ≥ 200 ng/ml, respectively. **(C)** OS rates of patients with high- or low- LARP1 expression when further divided into AFP < 100 ng/ml and AFP ≥ 100 ng/ml subgroups.

A validation cohort was employed to further evaluate the prognostic value of LARP1 for specific subgroups of patients. As shown in Figure 3, the LARP1 levels were significantly associated with OS in patients with early-stage HCC, and such a predictive power was also observed in patients without AFP elevation. In the subgroup of patients with low AFP (< 400 ng/ml), LARP1-low expression was associated with a 5-year OS rate of 54%, in contrast to 13% for the high-LARP1 group (*P* < 0.01, Figure 3A). Then we used different cutoff levels of AFP (400 ng/ml, 200 ng/ml and 100 ng/ml, respectively) to subgroup the 272 HCC patients and evaluated the prognostic significance of LARP1in the patient subgroups.Our data suggested that AFP cut-off values of 200 ng/ml or 100 ng/ml) were significantly predictive of patient survival, whereas AFP = 400 ng/ml was not a prognostic cut-off. However, in all above HCC patient subgroups, the level of LARP1 was more sensitive to predict the prognosis of HCC patients than AFP. As shown in the subgroup of AFP level less than 200 or 100 ng/ml, LARP1 was able to separate patients with different OS rates as well (Figure 3B and C).

**Figure 4:**
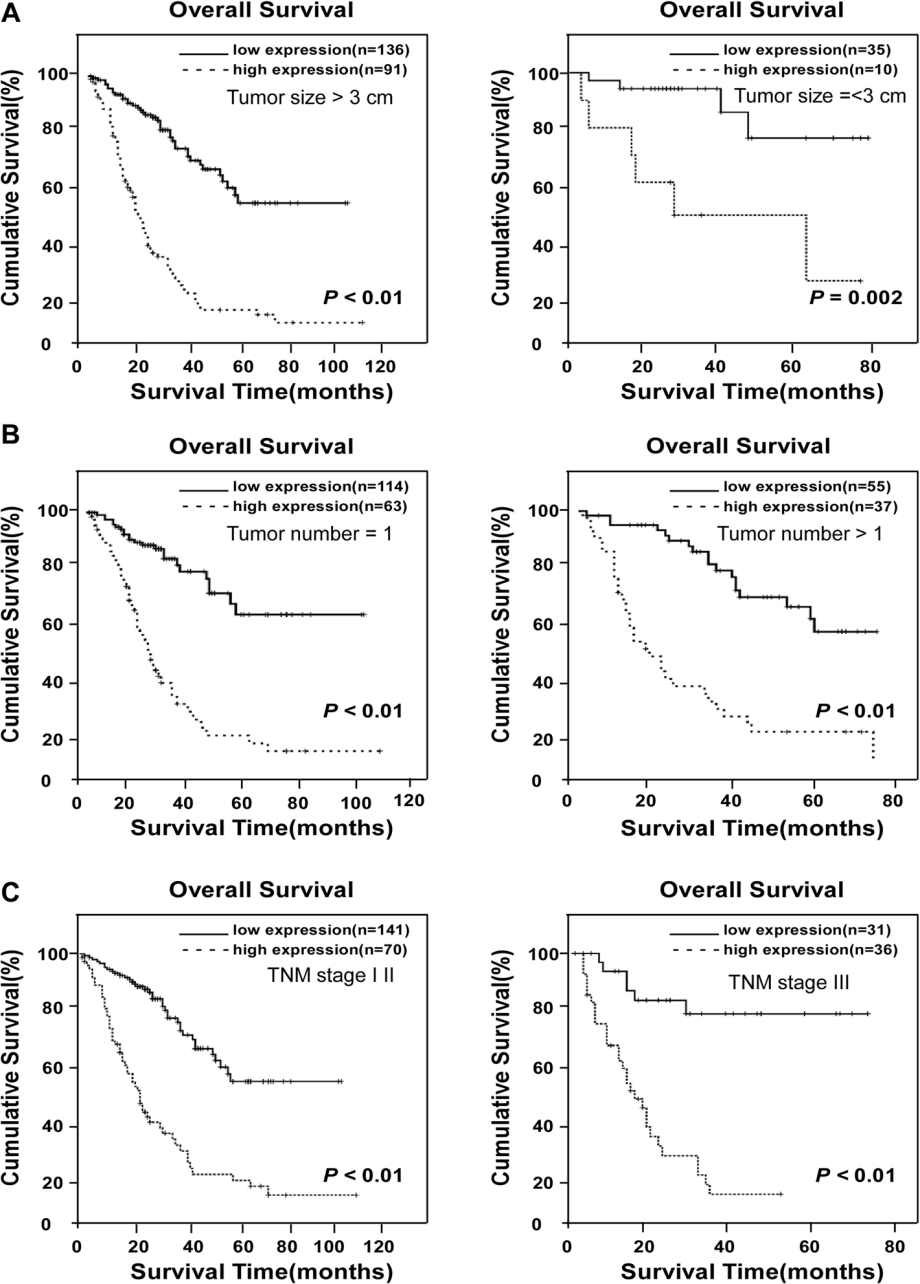
**Kaplan-Meier analysis of OS in 272 patients based on LARP1 expressions in HCC clinical subgroups. (A)** When patients were divided into subclinical groups according to tumor size, probabilities of survival with either HCC lesion diameter > 3 cm (left) or ≤3 cm (right), as well as high- and low-LARP1 expression, distinguishes lower and higher, respectively, SO rates. **(B)** OS in patients with single tumor lesion and multi-tumor lesion. **(C)** OS rates in patients subgrouped into TNM stages I-II (left) and TNM stages III (right) as differentiated by high- or low-LARP1 expression.

In another group of HCC patients whose survival has been difficult to predict in the clinic, namely, those with tumor sizes smaller than 3 cm in diameter, the 5-year survival rate was 75% in the low LARP1 group, as opposed to 23% for patients exhibiting high LARP1 expression (*P* = 0.005, Figure 4A). In the clinical subgroup with one single HCC tumor nodule, the 5-year survival rates were 63% and 9%, respectively, for low - or high -LARP1 expression patients (*P* < 0.01, Figure 4B). In early-stage HCC patients (TNM stages I-II), low -LARP1 expression patients revealed a 5-year survival rate of 53%, whereas the survival rate decreased to 13% in the high LARP1 group (*P* < 0.01, Figure 4C). Taken together, therefore, our data suggest a potentially promising prognostic value of LARP1 for HCC patients in various clinical subgroups that otherwise could have been difficult for survival prediction.

## Discussion

In the present study, a cohort of patients (n = 272) were examined for LARP1 expression. Consistent results derived from three different assays strongly suggest a correlation between LARP1 level and the clinical outcome of HCC. Identification of LARP1 as a prognostic biomarker for HCC in our present study provides new opportunities in the clinic for prediction of patient survival. The findings that high LARP1 expression level is present in HCC cell lines and clinical lesions, as well as its correlation with the clinical staging of HCC, lay a foundation for developing this immunologically and pathologically detectable molecule into a clinically applicable approach to improved patient management based on more accurate judgment on disease prognosis.

LARP1 is on chromosome 5q33.2 possesses a signature La motif, which is an ancient RNA-binding domain, plus a second conserved motif. LARP1 appears to bind RNA in vitro via both the La motif and the LARP1 domain [8]. Five LARP proteins exist in the human genome [9]. LARP1 regulates negatively RAS-MAPK pathway and is shown to be involved in cell division, migration and apoptosis [6,8]. LARP1 protein colocalizes with P bodies [8] which function in RNA degradation. But the role of LARP1 in HCC is unclear. Our current study employed large numbers of patients in independent cohorts (n = 272). The consistency of the results of three assay methods (RT-PCR, immunoblotting and immunohistochemistry) and the repeat validations in the test as well as the independent validation cohort, provide with essential, reliable evidence for the clinical significance of LARP1 as a prognostic biomarker for HCC.

AFP is most commonly employed in the clinic for HCC screening and as an important predictor for patient survival after tumor resection [10-12] The diagnostic sensitivity of AFP for early HCC, however, is only 39–64% when used alone, leading to the unsatisfying reality that a large number of HCC patients without AFP elevation are missed and subsequently progress to late stage-HCC before becoming clinically symptomatic and detectable [11]. Due to its low sensitivity in identifying new HCC cases that have not been detected by imaging technology previously, AFP has been shown to be only marginally effective in specific patient populations [11]. Indeed, only 38.0% of HCC patients in our study cohorts are AFP-positive. By contrast, in the patient group in which AFP level was not predictive for prognosis, LARP1 appeared to be indicative of survival time lengths that are differential among the patients. Thus, our study has exhibited the potential value of LARP1 in predicting patient survival in subgroups with normal AFP levels or in the early-stage HCC group, which would have been difficult using currently clinically available surrogate biomarkers.

Studies have shown that for HCC early diagnosis and treatment strategies, such as surgical resection and liver transplantation, there is a longer survival time associated with patients who have a single HCC tumor nodule < 3 cm in diameter, or with patients carrying no more than 3 nodules with each smaller than 3 cm in diameter, giving rise to a reported 5-year survival of 50 to 70% [13,14]. Using biomarkers to identify patients with a highest risk of developing worse prognosis may thus reduce mortality and medical costs. It is particularly noteworthy that in our test cohort, the patients with early HCC (TNM stages I-II) display a significantly higher levels (3–5 folds increase) of LARP1 in the HCC lesions than that in the normal liver tissue, and as the disease progresses to later stages, the LARP1 level increases further. In our validation cohort, early-stage (TNM stages I-II, tumor size less than 3 cm, single tumor nodule) HCC patients with low level of LARP1 protein immunostained also display a relative high OS than those diagnosed with late-stage HCC who carry high levels of LARP1 expression. In consistence with our observation, LARP1 has been mechanistically shown to promote cell invasion and metastasis [6]. High potential of vascular invasion and metastasis is often the main biological basis for the poor prognosis of HCC [14]. Thus, these characters of LARP1 thus warrant efforts to further explore the potential of LARP1 to become a promising biomarker for identifying patients with good prognosis after surgical excision in early stages. Inclusion of metastatic cases in future studies will help address whether a high-level expression of LARP1 in early-stage HCC patients may have the potential to progress to poor survival.

Several systems are available to classify HCC. Among them, the International Union Against Cancer’s TNM staging is one of the most prevalent. Although the TNM system has successfully graded patients on their prognosis according to clinicopathological variables, it is still limited in providing predictive information key to determining therapeutic strategies in subgroups of HCC patients, such as, prediction the prognosis of early-stage HCC. Thus, our current finding has provided evidence that the higher expression of LARP1 in HCC may be important for the detection of an aggressive phenotype or a phenotype predicting poor prognosis. We believe that the use of LARP1 protein as a diagnostic biomarker of HCC could improve the prospects of the early detection of HCC; and that an improved rate of detection would have important prognostic implications for patients with HCC. It is necessary to point out that our current study is of the retrospective nature and the number of patients with HCC lesions smaller than 3 cm was small. Clearly, further prospective studies designed to include a larger number of HCC lesions smaller than 3 cm and patients with metastasis are needed to validate the conclusions of this study. Moreover, it would be of great clinical value to determine the relationships of LARP1 with other signalling molecules and pathways which help us to better understand the molecular pathogenesis of these tumors and develop more effective targeted therapeutic strategies.

## Competing interests

The authors declare that they have no competing interest.

## Authors’ contributions

CX and SX designed and carried out most of the experiments and drafted the manuscript; DX, LH and GZ performed some experiments and provided administrative and technical support; PW, LH and LP performed patient data analysis and pathological examination; Z-lG contributed major ideas, proposed and designed the project, oversaw the project execution and revised the manuscript. All authors read and approved the final manuscript.
